# Higher Levels of Plasma Hyaluronic Acid and N-terminal Propeptide of Type III Procollagen Are Associated With Lower Kidney Function in Children With Non-alcoholic Fatty Liver Disease

**DOI:** 10.3389/fped.2022.917714

**Published:** 2022-06-06

**Authors:** Antonella Mosca, Alessandro Mantovani, Annalisa Crudele, Nadia Panera, Donatella Comparcola, Rita De Vito, Marzia Bianchi, Christopher D. Byrne, Giovanni Targher, Anna Alisi

**Affiliations:** ^1^Hepatology, Gastroenterology and Nutrition Unit, Bambino Gesù Children's Hospital, IRCCS, Rome, Italy; ^2^Section of Endocrinology, Diabetes and Metabolism, Department of Medicine, University and Azienda Ospedaliera Universitaria Integrata of Verona, Verona, Italy; ^3^Research Unit of Molecular Genetics of Complex Phenotypes, Bambino Gesù Children's Hospital, IRCCS, Rome, Italy; ^4^Unit of Pathology, Bambino Gesù Children's Hospital, IRCCS, Rome, Italy; ^5^Southampton National Institute for Health Research Biomedical Research Centre, Southampton General Hospital, University Hospital Southampton, Southampton, United Kingdom; ^6^Nutrition and Metabolism, Faculty of Medicine, University of Southampton, Southampton, United Kingdom

**Keywords:** children, NAFLD, kidney function, procollagen, hyaluronic acid

## Abstract

**Objective:**

Hyaluronic acid (HA) and N-terminal propeptide of type III procollagen (PIIINP) are two non-invasive biomarkers of liver fibrosis in non-alcoholic fatty liver disease (NAFLD). We examined the relationships of plasma levels of HA and PIIINP with kidney function in children with NAFLD.

**Methods:**

Plasma HA and PIIINP levels were measured using two commercially available enzyme-linked immunosorbent assay kits in a cohort of 106 Caucasian overweight or obese children with biopsy-proven NAFLD. Glomerular filtration rate (eGFR) was estimated using the Bedside Schwartz equation. Genotyping for the patatin-like phospholipase domain-containing protein-3 (*PNPLA3*) rs738409 variant was performed using an allelic discrimination assay.

**Results:**

Children with fibrosis F2 had significantly higher plasma PIIINP and HA levels than those with F0 or F1 fibrosis. Liver fibrosis was positively associated with plasma HA and PIIINP, as well as with the presence of the risk allele G of *PNPLA3* rs738409 variant, and negatively with eGFR. Moreover, eGFR showed significant inverse associations with HA and PIIINP levels, as well as the presence of G of *PNPLA3* rs738409, and liver fibrosis stage. Notably, our multivariable regression models showed that higher plasma PIIINP (standardized beta coefficient: −0.206, *P* = 0.011) and HA levels (standardized beta coefficient: −0.531, *P* < 0.0001) were associated with lower eGFR values, even after adjustment for age, sex, systolic blood pressure, *PNPLA3* rs738409 genotype, and any stage of liver fibrosis.

**Conclusions:**

Higher levels of HA and PIIINP were associated with lower eGFR values in Caucasian children with biopsy-proven NAFLD, independently of *PNPLA3* rs738409 genotype and other potential confounding factors.

## Introduction

Pediatric non-alcoholic fatty liver disease (NAFLD) has become an important public health problem in several developed countries, and with the exponential increase in childhood obesity, the estimated prevalence of NAFLD is near to 25% (1, 2). NAFLD is a multifactorial disease that encompasses simple steatosis, non-alcoholic steatohepatitis (NASH), advanced fibrosis and, ultimately, cirrhosis (3).

To date, accumulating evidence indicates that NAFLD is not only associated with adverse liver-related outcomes, but also with cardiovascular and kidney complications in both adults and children or adolescents (4–8). Several studies suggest that the relationship between NAFLD and chronic kidney disease could start in childhood (9). In particular, Targher et al. reported a significant association between patatin-like phospholipase domain-containing protein-3 (*PNPLA3*) rs738409 polymorphism (i.e., the major genetic variant associated with greater susceptibility to NAFLD development and progression) and kidney dysfunction in a pediatric population with histologically confirmed NAFLD. In this study the authors showed that the presence of the risk allele (G) of rs738409 was closely associated with decreasing estimated glomerular filtration rate (eGFR) values and increasing 24-h urinary protein excretion in Caucasian overweight children and adolescents with biopsy-proven NAFLD (10). Furthermore, Yodoshi et al. found that the renal impairment was significantly associated with liver disease severity in 179 children and adolescents with NAFLD. Twenty percent of these patients had glomerular hyper-filtration and 15% had a decreased eGFR within 3 months of their liver biopsy. Besides, glomerular hyper-filtration was associated with a higher histological NAFLD Activity score (NAS), independent of traditional renal risk factors, such as obesity, type 2 diabetes, and hypertension (11).

We hypothesized that concomitant liver and kidney disease in NAFLD could share common pathogenic mechanisms. We propose that liver and kidney disease could share common biomarkers of extracellular matrix (ECM) remodeling, such as increased hyaluronic acid (HA) and N-terminal propeptide of type III procollagen (PIIINP), which are two circulating biomarkers closely associated with the severity of liver fibrosis (12–14).

Hence, the main aim of our cross-sectional study was to evaluate the association of plasma HA and PIIINP levels with kidney dysfunction in children with biopsy-proven NAFLD.

## Methods

### Patients

A total of 106 Caucasian overweight or obese children and adolescents with ultrasound-defined hepatic steatosis (irrespective of serum aminotransferase levels) were enrolled to the Hepato-Metabolic Department of IRCCS “Bambino Gesù” Children's Hospital in Rome. All patients underwent liver biopsy for diagnosing and staging NAFLD. This practice was in agreement with the diagnostic flow chart proposed by the European Society for Pediatric Gastroenterology, Hepatology, and Nutrition Committee (15).

All children were tested to exclude secondary causes of hepatic steatosis, including alcohol consumption, total parenteral nutrition and chronic use of drugs known to induce hepatic steatosis (e.g., valproate, amiodarone, or prednisone). Hepatitis A, B, and C, cytomegalovirus, Epstein-Barr virus infections, and coeliac disease were also excluded using appropriate serological tests. Autoimmune liver disease, metabolic liver disease, Wilson's disease, and alpha-1-antitrypsin-associated liver disease were ruled out using standard clinical, laboratory, and histological criteria.

The study was carried out according to the rules of the Helsinki Declaration. Written informed consent was obtained from the parents of each child.

### Anthropometric, Clinical and Biochemical Measurements

Body mass index (BMI) and waist circumference (WC) were calculated as previously described (14). Blood pressure was measured in the right arm using a standard sphygmomanometer; and an average of three blood pressure values was reported. High blood pressure was defined by systolic or diastolic blood pressure >95th percentile for age, height and sex.

Venous blood samples were collected in the morning after an overnight fast of at least 8 h. Serum liver enzymes [aspartate aminotransferase (AST), alanine aminotransferase (ALT) and gamma-glutamyltransferase (GGT)], lipids [i.e., total cholesterol, high-density lipoprotein (HDL)-cholesterol, low-density lipoprotein (LDL)-cholesterol and triglycerides], glucose, insulin, high-sensitivity C-reactive protein (*hs-CRP*), uric acid, blood urea nitrogen and creatinine were measured in all children using standard laboratory procedures. The homeostasis model assessment-insulin resistance [HOMA-IR = insulin (mIU/L) x glucose (mmol/L)/22.5] was used to estimate a measure of insulin sensitivity. A cut-off value of HOMA-IR score >2.5 was considered as an index of insulin resistance (16). The estimated glomerular filtration rate (eGFR) was estimated using the Bedside Schwartz equation (eGFR = 0.413 x [height (cm)/serum creatinine (mg/dL)]), which has been documented to be accurate for estimating GFR in pediatric populations (17).

### Liver Histology

A diagnosis of NASH was established from liver biopsy, as previously reported (14). The characterized histological features of NAFLD were steatosis, portal and lobular inflammation, hepatocyte ballooning and fibrosis that were evaluated with the use of the scoring system developed by the National Institutes of Health-sponsored NASH Clinical Research Network (18). Steatosis was graded on a four-point scale: 0 = steatosis involving fewer than 5% of hepatocytes, 1 = steatosis involving up to 33% of hepatocytes, 2 = steatosis involving 33–66% of hepatocytes, and 3 = steatosis involving more than 66% of hepatocytes. Lobular inflammation was graded on a four-point scale: 0 = no foci, 1 = fewer than two foci per 200 × field, 2 = two to four foci per 200 × field, and 3 = more than four foci per 200 × field. Portal inflammation was graded on a four-point scale: 0 = none, 1 = mild, 2 = moderate, and 3 = severe. Hepatocyte ballooning was graded on a three-point scale: 0 = no ballooned cells, 1 = few ballooned cells and 2 = many/prominent ballooned cells. The stage of fibrosis was quantified using a five-point scale: 0 = no fibrosis, 1 = peri-sinusoidal or periportal fibrosis [(1a) mild, zone 3, perisinusoidal; (1b) moderate, zone 3, perisinusoidal; and (1c) portal/periportal], 2 = peri-sinusoidal and portal/periportal fibrosis, 3 = bridging fibrosis, and 4 = cirrhosis (19). The NAFLD activity score (NAS) was defined as the sum of the scores for steatosis, lobular inflammation, and ballooning. Cases with NAS of 3 and 4 were diagnosed as borderline NASH, whereas cases with scores of ≥5 were diagnosed as definite NASH. This diagnosis was reviewed and confirmed by an expert pathologist (RDV). Histological characteristics of children with NAFLD are reported in Supplementary Table 1.

### PIIINP and HA Measurements

For assessment of plasma PIIINP and HA levels, 3–4 ml of venous blood samples were collected in EDTA buffered tubes by each subject after an overnight fast. Blood samples were centrifuged at 2,000 g by 10 min using a refrigerated centrifuge and plasma samples were stored at −80°C. Plasma concentrations of HA and PIIINP were measured by commercially available ELISA kits (Hyaluronan Quantikine ELISA, R&D, code: DHYAL0, R&D Systems, Minneapolis, MN, United States; Human Procollagen type III N-terminal Propeptide ELISA Kit, code: NBP2-76434, Centennial, CO, United States), according to the manufacturer's instructions.

### PNPLA3 Genotyping

The rs738409 C>G polymorphism in *PNPLA3* gene has been genotyped by TaqMan 5'-nuclease (Applied Biosystems, Foster City, CA, United States) by staff unaware of the clinical status of patients. Briefly, genomic DNA was isolated from venous blood using a Blood DNA Extraction Kit (Qiagen, Valencia, CA, United States). The absorbance ratio at 260/280 nm of all the samples ranged from 1.8 to 2 indicating they were all free from contaminants. Real-time PCR was performed using Applied Biosystems 7900HT Fast Real-Time PCR System (Applied Biosystems, Carlsbad, CA, United States). Positive and negative controls were included on each reaction plate to verify the reproducibility of the results.

### Statistical Analysis

Data are expressed as means ± SD, medians and interquartile ranges (IQR), or percentages. Differences in anthropometric, clinical and biochemical characteristics among the patient groups were tested by using the Fisher's exact test for categorical variables, the one-way ANOVA for normally distributed continuous variables, or the Kruskal-Wallis test for non-normally distributed continuous variables. Pearson's correlation coefficients were calculated to examine univariable linear associations between plasma HA or PIIINP levels (after logarithmic transformation) and liver fibrosis or eGFR values.

Mann-Whitney *U*-test with Bonferroni adjustment was used to compare differences between independent samples. The medians for continuous parameters and the values si = 1 and no = 0 for ordinals were considered.

Subsequently, multivariable linear regression modeling was used to test the independence of associations between these two circulating biomarkers as key exposures, with eGFR values as the key outcome (as a continuous measure), after adjusting for potential confounding factors, such as age, sex, systolic blood pressure, *PNPLA3* rs738409 genotype, and presence of any stage of liver fibrosis. Covariates included in all multivariable regression models were selected as potential confounders based on their significance in univariable regression analyses or based on their biological plausibility.

Statistical analyses were performed using STATA software, version 14.2 (STATA, College Station, TX, United States). A *P* < 0.05 was considered statistically significant.

## Results

We evaluated a cohort composed of 106 overweight and obese children with biopsy-proven NAFLD. Main clinical and biochemical characteristics of these children are reported stratified by increasing eGFR tertiles in Supplementary Table 2. Next, we also evaluated the anthropometric, biochemical and clinical characteristics of these children, stratified by increasing stages of liver fibrosis. Specifically, as shown in Table 1, age, sex, body weight, BMI, blood pressure, total and LDL-cholesterol, fasting glucose, insulin, HOMA-IR, hs-CRP, and liver enzymes did not differ significantly among the three groups of children. On the contrary, WC, HDL-cholesterol, triglycerides, uric acid, blood urea nitrogen, creatinine, and eGFR were significantly different between the three patient groups (*P* ≤ 0.05). Of note, as shown in Table 2, children with NAFLD and fibrosis *F* ≥ 2 also had markedly higher levels of plasma PIIINP and HA than those with fibrosis F0 or F1 (*P* < 0.001 for the trend). In addition, children with fibrosis *F* ≥ 2 also had a higher prevalence of the genotype GG and CG *PNPLA3* rs738409 (*P* = 0.0048).

**Table 1:** Main anthropometric and clinical characteristics of children with biopsy-confirmed NAFLD, stratified by increasing histological stages of liver fibrosis.

	**F0 (*n* = 16)**	**F1 (*n* = 69)**	**F2 (*n* = 21)**	***P*-value**
Age (years)	12.5 ± 2.5	11.5 ± 2.8	12.4 ± 3.1	0.269
Male sex (%)	75	51.6	57.1	0.626
Weight (kg)	67.5 ± 19.6	70.5 ± 17.4	70.2 ± 22.3	0.163
BMI (kg/m^2^)	27.5 ± 6.1	27 ± 5.1	27.8 ± 4.2	0.350
WC (cm)	85.3 ± 15	84.5 ± 14.3	87.6 ± 12.7	**0.002**
Systolic blood pressure (mmHg)	112 ± 12.3	112.7 ± 15.1	114.3 ± 12.9	0.146
Diastolic blood pressure (mmHg)	58.3 ± 13	62.7 ± 9.6	60.3 ± 11.9	0.057
Total cholesterol (mg/dl)	163 ± 31	153 ± 27.8	152 ± 29.4	0.051
LDL-cholesterol (mg/dl)	103 ± 27.4	98 ± 25.9	99 ± 27.3	0.057
HDL-cholesterol (mg/dl)	45 ± 14.2	45 ± 8.6	42 ± 8.1	**0.014**
Triglycerides (mg/dl)	96 (23–168)	109 (59–125)	109.2 (56–161)	**0.036**
Fasting glucose (mg/dl)	88.4 ± 19.8	83.6 ± 9.8	80.3 ± 8.4	0.052
Fasting insulin (mIU/L)	17.5 (6.5–27.6)	16.4 (12–21.3)	19.8 (10.3–29.4)	0.098
HOMA-IR score	3.7(1.2–5.6)	3.4 (2.3–4.9)	3.9 (1.9–6.4)	0.674
hs-CRP (mg/L)	0.23 ± 0.31	0.3 ± 0.46	0.29 ± 0.5	0.816
Uric acid (mg/dl)	5.3 ± 1.5	5.4 ± 1.25	6.2 ± 1.8	**0.004**
Urea nitrogen (mg/dl)	14.7 ± 2.3	14.3 ± 2.9	14.3 ± 3.4	**0.003**
Creatinine (mg/dl)	0.53 ± 0.08	0.56 ± 0.12	0.61 ± 0.11	**<0.001**
eGFR_Bedside−Schwartz_ (ml/min/1.73 m^2^)	165.7 ± 26.2	150.3 ± 24	140.6 ± 24.1	**<0.001**
AST (IU/L)	27 (16–31)	27 (24–35)	26 (19–31)	0.529
ALT (IU/L)	27 (14–34)	29 (20–50)	29 (19–61)	0.670
GGT (IU/L)	15 (8–17)	15 (12–21)	16 (13–22)	0.590

*Data are expressed as means ± SD or medians and interquartile ranges (in parenthesis), or percentages. Differences among the three groups of children were tested by the Fischer's exact test for categorical variables, the one-way ANOVA for normally distributed continuous variables, or the Kruskal–Wallis test for non-normally distributed continuous variables*.

*BMI, body mass index; WC, waist circumference; LDL, low-density lipoprotein; HDL, high-density lipoprotein; HOMA-IR, homeostasis model assessment-insulin resistance; hs-CRP, high-sensitivity C-reactive protein; eGFR, glomerular filtration rate; AST, aspartate aminotransferase; ALT, alanine aminotransferase; GGT, gamma-glutamyltransferase. The bold values indicate the significant p values*.

**Table 2:** Biomarkers of extracellular matrix remodeling and genetic parameters of children with biopsy-confirmed NAFLD, stratified by increasing histological stages of liver fibrosis.

	**F0 (*n* = 16)**	**F1 (*n* = 69)**	**F2 (*n* = 21)**	***P-*value**
HA (ng/ml)	25.2 (18.2–30.7)	35.6 (26.1–54)	172 (187–209)	**<0.001**
PIIINP (ng/ml)	3.9 (0.8–5.6)	5.5 (3.5–8.2)	9.56 (7.2–11.4)	**<0.001**
PNPLA3 rs738409				**0.005**
CC genotype (%)	43.0	28.9	57.1	
CG genotype (%)	50.0	39.2	23.8	
GG genotype (%)	6.2	31.9	19.1	

*Data are expressed as medians and interquartile ranges (in parenthesis), or percentages. Differences among the three groups of children were tested by the Fischer's exact test for categorical variables, the one-way ANOVA for normally distributed continuous variables, or the Kruskal-Wallis test for non-normally distributed continuous variables*.

*HA, hyaluronic acid; PIIINP, N-terminal propeptide of type III procollagen*.

As shown in Table 3, there was a negative correlation between stages of fibrosis and eGFR values (*r* = −0.22; *P* = 0.03). Furthermore, increasing fibrosis stage (*F* ≥ 1) was positively correlated with levels of both HA (*r* = 0.56; *P* = 0.0001) and PIIINP (*r* = 0.38; *P* = 0.0001), as well as with the presence of the risk allele G of *PNPLA3* rs738409 variant (*r* = 0.28; *P* = 0.003). In contrast, eGFR values were negatively correlated with levels of HA (*r* = −0.42; *P* < 0.0001) and PIIINP (*r* = −0.33; *P* = 0.006), as well as with the presence of the risk allele G of *PNPLA3* rs738409 variant (*r* = −0.33; *P* = 0.006).

**Table 3:** Univariate associations between clinical/biochemical and genetic variables with levels of liver fibrosis or eGFR, in children with biopsy-proven NAFLD.

	**Liver fibrosis**		**eGFR**	
	***r***	***P*-value**	***r***	***P*-value**
BMI	0.02	0.79	−0.12	0.24
Creatinine	0.18	0.06	−0.76	**<0.0001**
Triglycerides	−0.09	0.83	−0.19	0.07
Total cholesterol	−0.10	0.28	−0.18	0.06
Uric acid	0.18	0.07	−0.15	0.10
HOMA-IR score	−0.16	0.09	0.09	0.45
ALT	0.18	0.06	−0.04	0.67
eGFR_BedsideSchwartz_	−0.22	**0.03**	ND	–
HA	0.56	**0.0001**	−0.42	**<0.0001**
PIIINP	0.38	**0.0001**	−0.33	**0.006**
PNPLA3 (CG + GG)	0.28	**0.003**	−0.33	**0.006**
PNPLA3 GG	0.31	**0.004**	−0.36	**0.005**
PNPLA3 CG	0.26	**0.03**	−0.24	**0.048**
PNPLA3 CC	0.19	**0.12**	−0.20	**0.07**
Fibrosis stage	ND	–	−0.22	**0.03**

*BMI, body mass index; HOMA-IR, homeostasis model assessment-insulin resistance; eGFR, glomerular filtration rate; ALT, alanine aminotransferase; HA, hyaluronic acid; PIIINP, N-terminal propeptide of type III procollagen; ND, not determined. The bold values indicate the significant p values*.

The *U*-test also showed (Supplementary Table 3) that PNPLA3-CG+GG, PIIINP and HA are also associated with fibrosis (z-score = 2.01, = 2.88; = 3.11; *P* < 0.05), while PIIINP and HA are also associated with portal inflammation (z-score = 5.8, = 5.5, *P* < 0.05).

In Table 4 are reported the independent associations between either plasma PIIINP or HA levels and eGFR values (included as a continuous measure) after adjusting for the risk allele G of *PNPLA3* rs738409 variant, any stage of liver fibrosis and other potential confounding factors.

**Table 4:** Independent associations between eGFR values (included as a continuous measure) and plasma levels of either PIIINP (model 1) or HA (model 2), adjusted for age, sex, systolic blood pressure, *PNPLA3* rs738409 genotype, and stages of liver fibrosis in children with NAFLD.

**Linear regression analysis**	**Std. beta coefficient**	**SE**	***P*-value**
**eGFR**_**BedsideSchwartz**_ **(ml/min/1.73 m**^**2**^**)**			
**Adjusted model 1**			
PIIINP (ng/ml)	−0.206	0.65	**0.011**
Age (years)	−0.222	0.88	0.066
Sex (M vs. F)	−0.027	4.7	0.798
Systolic blood pressure (mmHg)	0.128	0.20	0.306
**PNPLA3 rs738409**			
CC genotype (*n* = 31)	*Ref*.		
CG genotype (*n* = 40)	−0.204	2.7	0.107
GG genotype (*n* = 35)	−0.334	3.0	**0.021**
Liver fibrosis, any stage (F0 vs. F ≥ 1)	−0.018	4.5	0.883
**Adjusted model 2**			
HA (ng/ml)	−0.531	0.57	**<0.0001**
Age (years)	−0.185	0.85	0.101
Sex (M vs. F)	0.064	4.4	0.517
Systolic blood pressure (mmHg)	0.181	0.18	0.202
**PNPLA3 rs738409**			
CC genotype (*n* = 31)	*Ref*.		
CG genotype (*n* = 40)	−0.191	2.4	0.108
GG genotype (*n* = 35)	−0.248	3.1	0.068
Liver fibrosis, any stage (F0 vs. F ≥ 1)	0.160	4.7	0.204

*Data are expressed as standardized beta coefficients as tested by multivariable linear regression analysis*.

*eGFR, glomerular filtration rate; PIIINP, N-terminal propeptide of type III procollagen; HA, hyaluronic acid, Ref., reference category. The bold values indicate the significant p values*.

In adjusted model 1, we found that increasing plasma PIIINP levels (included as logarithmically transformed variable) were associated with lower eGFR values (standardized beta coefficient: −0.206; *P* = 0.01), independent of age, sex, *PNPLA3* rs738409 genotype and histologic stage of liver fibrosis (F0 vs. F1 ≥ 1). In this model, the GG *PNPLA3* rs738409 genotype was also independently associated with lower eGFR values. Conversely, in the adjusted model 2, only increasing plasma HA levels were significantly associated with lower eGFR values (standardized beta coefficient: −0.531; *P* < 0.0001) after adjustment for the aforementioned covariates.

## Discussion

Our observational study reports for the first time a significant association between higher plasma HA or PIIINP levels and lower kidney function parameters in a pediatric NAFLD population. In particular, the main finding of our study was that plasma levels of both HA and PIIINP were negatively associated with eGFR_BedsideSchwartz_ values.

While the role of circulating levels of HA and PIIINP as biomarkers of liver fibrosis has been well established in children with NAFLD (14, 20, 21), the specific contribution of these two biomarkers in predicting lower eGFR values in a setting of pediatric NAFLD has not been investigated. HA is a high molecular weight glycosaminoglycan with linear polysaccharide structure that is synthesized by several cell types, including fibroblasts (22). Over the last decade, it has become increasingly evident that circulating levels of HA are strongly related to the severity of liver fibrosis in patients with various chronic liver diseases (including NAFLD), probably owing to the increased extracellular matrix deposition in hepatic fibrosis and the reduced clearance of HA by sinusoidal endothelial cells in the liver (23). Increased levels of HA have been found in renal tissue and serum in both experimental models and human samples of several kidney diseases (24, 25). Moreover, in a case-control study of 164 critically ill adult patients and 61 healthy controls, Yagmur et al. (26) reported that circulating HA levels were associated with impaired kidney parameters (i.e., eGFR and cystatin C) and liver function (i.e., albumin and pseudocholinesterase). Our study confirms these findings in a cohort of overweight or obese children and/or adolescents with biopsy-confirmed NAFLD, showing that higher plasma levels of HA were closely associated with lower values of eGFR_BedsideSchwartz_. The independent and inverse association we observed between plasma HA levels and eGFR in pediatric NAFLD might have two possible explanations. Firstly, as HA is involved in regulating the development of inflammation and fibrosis in the kidney, the increase in circulating levels of this biomarker might represent a trait of ECM remodeling occurring during development and progression of kidney disease (27). Alternatively, as suggested by Pecoits-Filho et al. (28), as HA is partially cleared by the kidney, circulating levels of this biomarker might reflect renal dysfunction.

PIIINP is a procollagen III cleavage product that can be used as a circulating biomarker of ECM remodeling during liver fibrogenesis (29, 30), but a role for this molecule in kidney disease has also been reported in adult patients with various stages of chronic kidney disease (CKD) (31). In that study, Ghoul et al. (31) documented that a higher urinary PIIINP/creatinine ratio (UPIIINP/Cr) was associated with lower eGFR values, higher CKD stage, and greater interstitial renal fibrosis determined by kidney histology. Subsequently, other studies showed that higher PIIINP levels, both in urine and serum, were associated with increasing stages of CKD and with interstitial renal fibrosis in both adults and children (32, 33). According with these findings, we have shown that higher levels of plasma PIIINP were strongly associated with lower eGFR values in our children with NAFLD, even if the strength of this association was slightly lower than that observed for HA. A potential mechanistic explanation for this association might also be linked to increased ECM turnover in the damaged organ. This may occur as a result of breakdown of renal interstitial collagen III by multiple collagenolytic enzymes, released by infiltrating inflammatory cells as impaired renal function occurs (32). Future mechanistic studies are required to better understand the precise molecular mechanisms underpinning the association between plasma HA and PIIINP levels and kidney dysfunction in NAFLD. Moreover, it could be informative to investigate other surrogate markers, such as lumican, which may provide a direct measure of ECM protein remodeling rate and collagen turnover occurring in the fibrotic liver (34).

Finally, we found that the major risk *PNPLA3* allele G for NAFLD was also associated with higher liver fibrosis stage and lower eGFR, thereby confirming observations from previous studies, showing the potential impact of this genetic polymorphism on the link between NAFLD and CKD (35).

Our study has some important limitations that should be mentioned. Firstly, this study has a cross-sectional design that does not allow us to establish the temporality and causality of the observed associations. Secondly, it is possible that the single ethnicity of our study participants may cause selection bias; thus our results might not be generalizable to other pediatric populations of different ethnicity. Thirdly, we were unable to directly measure GFR (by plasma iohexol disappearance) and, therefore, we used the most widely accepted prediction formula for estimating GFR in our pediatric population (i.e., the Bedside Schwartz formula) (17). Despite these limitations, our study has several important strengths, including the relatively large sample size, the consecutive enrolment of participants, the database completeness, and the use of liver biopsy to diagnose and stage NAFLD.

In conclusion, our study shows for the first time that higher circulating levels of HA and PIIINP are significantly associated with lower eGFR values in overweight or obese children with biopsy-proven NAFLD. Notably, these associations remained significant even after adjustment for common renal risk factors, presence of liver fibrosis and the *PNPLA3* rs738409 genotype.

## Data Availability Statement

The original contributions presented in the study are included in the article/Supplementary Material, further inquiries can be directed to the corresponding author/s.

## Ethics Statement

The studies involving human participants were reviewed and approved by Bambino Gesù Children's Hospital Ethics Commitee. Written informed consent to participate in this study was provided by the participants' legal guardian/next of kin.

## Author Contributions

AA, GT, and CB contributed to study design, data interpretation, wrote the manuscript, and reviewed and edited the manuscript. AMo, AMa, AC, NP, and MB contributed to the analysis and interpretation of data and wrote the manuscript. DC and RD contributed to data collection and clinical interpretation. All authors approved the final submitted version of the manuscript.

## Funding

This study was supported by the 5x1000 2022 to AA; CB was supported in part by the Southampton NIHR Biomedical Research Center (IS-BRC-20004), United Kingdom. GT was supported in part by grants from the University School of Medicine of Verona, Verona, Italy.

## Conflict of Interest

The authors declare that the research was conducted in the absence of any commercial or financial relationships that could be construed as a potential conflict of interest.

## Publisher's Note

All claims expressed in this article are solely those of the authors and do not necessarily represent those of their affiliated organizations, or those of the publisher, the editors and the reviewers. Any product that may be evaluated in this article, or claim that may be made by its manufacturer, is not guaranteed or endorsed by the publisher.
