# Supplementary material for: Higher Levels of Plasma Hyaluronic Acid and N-terminal Propeptide of Type III Procollagen Are Associated With Lower Kidney Function in Children With Non-alcoholic Fatty Liver Disease
Source: Front Pediatr. 2022 Jun 6;10:917714. doi: 10.3389/fped.2022.917714 (PMC9207333; doi:10.3389/fped.2022.917714)
Supplement: Supplementary file 1 [file Table_1.pdf]

Supplementary Table 1. Histological characteristics of children with biopsy-confirmed NAFLD.

	***n*=106**
Steatosis grade	
0	0 (%)
1	26 (24.5%)
2	47 (44.3%)
3	33 (31.2%)
Lobular inflammation	
0	11 (10.4%)
1	72 (67.9%)
2	23 (21.7%)
Portal inflammation	
0	8 (7.5%)
1	76 (71.7%)
2	22 (20.8%)
Ballooning degeneration	
0	17 (16%)
1	44 (41.5%)
2	45 (42.5%)
Fibrosis stage	
0	16 (15.2%)
1	69 (65%)
2	21 (19.8%)
3	0 (0%)
**Definite NASH**	68 (64.1%)

Supplementary Table 2. Clinical and biochemical characteristics of children with biopsy-proven NAFLD, stratified by tertiles of eGFR $_{\text{Bedside Schwartz}}$.

**Parameters**	**1st tertile (*n*=36)**	**2nd tertile (*n*=35)**	**3rd tertile (*n*= 35)**	**p value**
Age (years)	12.6 ± 3.0	11.3 ± 3.0	11.5 ±2.4	0.114
Male sex (%)	47.2	65.7	51.4	0.271
Weight (kg)	67.5 ± 24.3	60.5 ± 19.4	64.2 ± 19.9	0.382
BMI (kg/m²)	28.4 ± 5.2	26.1 ± 4.0	27.2 ± 5.7	0.161
WC (cm)	88.5 ± 13.3	83.8 ± 13.6	83.2 ± 15.1	0.219
Systolic blood pressure (mmHg),	116 ± 12	108 ± 14	114 ± 16	0.101
Diastolic blood pressure (mmHg),	60 ± 10	62 ± 11	63 ± 11	0.662
Total cholesterol (mg/dL)	161 ± 25.4	152 ± 25.9	150 ± 33.4	0.213
LDL-cholesterol (mg/dL)	104 ± 22.3	99 ± 26.3	93 ± 30.0	0.282
HDL-cholesterol (mg/dL)	44 ± 10.8	44 ± 7.9	44 ± 9.8	0.997
Triglycerides (mg/dL)	99 (64-172)	88 (64-110)	70 (52-129)	0.219
Fasting glucose (mg/dL)	83.0 ± 8.9	82.8 ± 9.5	85.2 ± 15.9	0.652
Fasting insulin (mIU/L)	15.9 (11.0-26.9)	15.0 (9.6-21.3)	17.0 (10.0-22.2)	0.608
HOMA-IR score	3.5 (2.2-5.9)	3.3 (1.9-4.9)	3.7 (2.6-5.3)	0.628
hs-CRP (mg/L)	0.11 (0.05-0.25)	0.09 (0.05-0.31)	0.05 (0.05-0.31)	0.709
Blood urea nitrogen (mg/dL)	14.6 ± 3.3	14.8 ± 2.8	13.6 ±2.7	0.203
Creatinine (mg/dL)	0.67 ± 0.11	0.55 ± 0.07	0.46 ± 0.06	**<0.001**
eGFR$_{Bedside\ Schwartz}$ (mL/min/1.73 m²)	94 ± 7	114 ± 5	139 ± 15	**ND**
Proteinuria (mg/24h)	50 (33-70)	48 (38-56)	50 (37-66)	0.797
Albuminuria (mg/24h)	4.7 (2.7-10.3)	4.1 (2.7-6.8)	4.2 (3.3-8.9)	0.723
AST (IU/L)	26 (24-32)	30 (24-35)	27 (23-32)	0.567
ALT (IU/L)	26 (20-34)	32 (21-48)	25 (19-49)	0.750
GGT (IU/L)	15 (11-19)	16 (14-23)	14 (11-20)	0.354
PIIINP (ng/mL)	8.30 (5.4-12.0)	5.51 (3.8-8.8)	4.61 (2.2-7.4)	**0.003**
HA (ng/mL)	73.8 (38.3-179.0)	37.3 (28.4-76.0)	28.0 (21.0-36.4)	**<0.001**
Definite NASH (%)*	41.7	45.7	48.6	0.857
PNPLA3 rs738409				**0.030**
CC genotype (%)	11.1	40.0	37.1	
CG genotype (%)	44.4	28.6	40.0	
GG genotype (%)	44.4	31.4	22.9	

Data are expressed as means ± SD, medians and interquartile ranges (in parenthesis), or percentages.
**Definite NASH was defined as presence of NAFLD Activity Score [NAS] ≥5 on histology.*

Abbreviations: BMI, body mass index; WC, waist circumference; LDL, low-density lipoprotein; HDL, high-density lipoprotein; HOMA-IR, homeostasis model assessment-insulin resistance; hs-CRP, high-sensitivity C-reactive protein; eGFR, glomerular filtration rate; AST, aspartate aminotransferase; ALT, alanine aminotransferase; GGT, gamma-glutamyltransferase; PIIINP, N-terminal propeptide of type III procollagen; HA, hyaluronic acid; NASH, non-alcoholic steatohepatitis; ND, not determined.

Supplementary Table 3. Mann-Whitney U-test comparisons between HA, PIINP, and PNPLA3 genotype and histological features.

	**Steatosis**		**Lobular inflammation**		**Portal inflammation**		**Ballooning**		**Fibrosis**		**NAS**	
	z	***p***	***z***	***p***	***z***	***p***	***z***	***p***	***z***	***p***	***z***	***p***
HA	2.88	0.01	4.70	0.01	5.5	0.012	1.91	0.11	3.11	0.02	-2.78	0.002
**PIIINP**	-5.51	0.001	-2.82	0.001	5.8	0.001	-1.79	0.08	2.88	0.001	2.67	0.001
PNPLA3 *(CG+GG)*	3.26	0.01	-2.23	0.024	-1.4	0.16	-1.51	0.09	2.01	0.044	-5.52	0.001

Abbreviations: HA, hyaluronic acid; PIIINP, N-terminal propeptide of type III procollagen; NAS, NAFLD activity score.
